# Pseudohypoparathyroidism: Challenges in Early Recognition and Diagnosis of a Rare Hereditary Disorder

**DOI:** 10.7759/cureus.79321

**Published:** 2025-02-19

**Authors:** Joana Glória, Mariana Soares, Marta P Soares, Carla Pereira, Maria de Lurdes Sampaio

**Affiliations:** 1 Pediatrics, Unidade Local de Saúde Santa Maria, Lisbon, PRT; 2 Genetics, Unidade Local de Saúde Santa Maria, Lisbon, PRT; 3 Pediatric Endocrinology, Unidade Local de Saúde Santa Maria, Lisbon, PRT

**Keywords:** congenital hypothyroidism, gnas gene mutation, hyperphosphatemia, hypocalcaemia, pseudohypoparathyroidism

## Abstract

Pseudohypoparathyroidism (PHP) is an uncommon endocrine condition marked by an impaired response to parathyroid hormone (PTH), which results in biochemical abnormalities. Clinical manifestations can vary significantly, occasionally resembling other endocrine disorders. Genetic testing plays a critical role in distinguishing PHP from other conditions, as it enables precise diagnosis even when classical features are not initially present.

We report the case of a male infant initially diagnosed with congenital hypothyroidism (CH) through neonatal screening and treated with levothyroxine. While growing up, the patient developed syndromic features, including facial dysmorphisms, global developmental delay, behavioural issues, and early-onset obesity. Whole exome sequencing (WES), prompted by the complex phenotype, identified a maternally inherited *GNAS* variant. This led to the suspicion of PHP1A, which was subsequently confirmed at five years of age when laboratory reevaluation revealed hypocalcemia, hyperphosphatemia, and elevated PTH levels. PHP can mimic isolated CH in early presentations, often delaying recognition.

This case underscores the pivotal role of genetic testing in diagnosing PHP. Early genetic evaluation and multidisciplinary care are essential for accurate diagnosis, tailored treatment, and long-term monitoring, ultimately improving patient outcomes.

## Introduction

Pseudohypoparathyroidism (PHP) is a rare hereditary disorder that impairs the ability to respond to parathyroid hormone (PTH), even when the hormone levels are in the normal range. PTH is essential for calcium and phosphorus homeostasis, working with vitamin D to regulate their metabolism in the blood and bones. When the body's response to PTH is impaired, it disrupts calcium homeostasis, leading to hypocalcemia, hyperphosphatemia, and potential skeletal abnormalities. This hormonal resistance can manifest clinically through muscle cramps, seizures, and characteristic physical features such as short stature, round facial appearance, and shortened fingers (brachydactyly) [1,2].

The molecular basis of PHP adds another layer of complexity, as genetic testing frequently identifies pathogenic variants in genes such as GNAS, and STX16 but also PDE4D and PRKAR1A. Variants in GNAS lead to a deficiency of the alpha subunit of the stimulatory G protein (Gsα), impairing G protein-coupled receptor signalling. This subunit is encoded in GNAS exons 1-13 [3], usually expressed from both maternal and paternal alleles in most cells. However, in certain cells (such as pituitary somatotropes, kidney tubule cells, thyroid cells and gonadal cells), Gsα protein is mainly expressed from the maternal allele [3]. Thus, pathogenic variants in the maternal allele affect the function of multiple hormones [1-3]. Consequently, in addition to classic PTH resistance, PHP can also disrupt other hormonal pathways, such as those involving thyroid-stimulating hormone (TSH), potentially resulting in hypothyroidism [1-4].

There are five PHP subtypes, with the most common being PHP subtype 1A (PHP1A) and PHP subtype 1B (PHP1B). PHP1A is caused by maternal loss-of-function variants in the GNAS gene coding sequence, while PHP1B results from methylation defects at the GNAS locus [5]. The diverse and complex genetic and epigenetic abnormalities underlying these disorders require a specialized approach to achieve an accurate molecular diagnosis, which can often be challenging [5]. Distinguishing between subtypes is crucial, as clinical presentation is sometimes non-specific and non-differentiating, making genetic diagnosis essential.

Clinical presentations of PHP are highly variable, ranging from muscle spasms and seizures to the distinct features of Albright hereditary osteodystrophy (AHO), including short stature, brachydactyly, obesity, a rounded face, heterotopic ossification, and cognitive impairment. AHO is a key phenotypic marker of certain PHP subtypes, particularly PHP1A [1,5], and its presence can aid in the clinical suspicion of PHP. Early recognition is further complicated when hypocalcemia or overt physical characteristics are absent [1-3], which results in many cases remaining undiagnosed.

Diagnosing PHP is additionally challenging not only due to its subtle clinical manifestations but also because of its overlap with more common conditions, like congenital hypothyroidism (CH). While PHP affects 0.34-1.1 per 100,000 individuals [6], CH is significantly more prevalent, occurring in 1 in 2,000 to 1 in 4,000 newborns [7]. In infants, PHP is often misdiagnosed as isolated CH, through newborn screening [2,6]. As mentioned, the dysregulation in PHP can affect various hormonal pathways, including those involving TSH, which helps explain this overlap.

This case report on PHP emphasizes the need for early recognition and diagnosis due to its diverse and complex clinical presentation and potential for misdiagnosis. Despite its low prevalence, PHP serves as a valuable model for understanding hormone resistance syndromes, which can lead to a range of complications, including hypocalcemia, skeletal deformities, cognitive impairment, and other endocrine dysfunctions. By highlighting the challenges in early diagnosis and the need for clinical suspicion, this report aims to improve awareness among clinicians and enhance diagnostic accuracy in pediatric populations.

## Case presentation

This case report describes a male infant born at 35 weeks of gestation, weighing 2570g (appropriate for gestational age). He experienced neonatal jaundice requiring phototherapy. The family history included a deceased sister at nine days of age, with the cause of death undetermined. Suspicion of CH was raised through the National Neonatal Screening Program, with TSH levels of 10.7 µIU/mL and total T4 levels of 8.6 µg/dL, leading to his referral to a pediatric endocrinology clinic at a tertiary reference centre. Laboratory analysis confirmed the diagnosis (TSH 19.2 µIU/mL, total T4 7.3 µg/dL, free T4 (fT4) 0.88 ng/dL; Table 1) and levothyroxine therapy was started.

**Table 1:** Laboratory tests

	Neonatal Screening	Reference range	1 month old	Reference range	5 months old	Reference range	5 years old	Reference range
TSH (µIU/mL)	10.7	[1.2-10.7]	19.2	[0.6-7.3]	0.3	[0.6-7.3]	8.94	[0.7-6.6]
Total T4 (µg/dL)	8.6	[8-21.8]	7.3	[7.2-15.7]	10.2	[7.2-15.7]	6.4	[6.4-13.5]
fT4 (ng/dL)	-	-	0.88	[0.9-2.3]	1.46	[0.9-2.3]	0.83	[0.8-1.8]
Calcium (mg/dL)	-	-	-	-	10.0	[8.5-11.0]	7.0	[8.5-11.0]
Phosphorus (mg/dL)	-	-	-	-	5.5	[3.5-6.6]	8.5	[3.5-6.6]
Magnesium (mg/dL)	-	-	-	-	2.4	[1.7-2.3]	2.2	[1.7-2.3]
PTH (pg/mL)	-	-	-	-	40.2	[14-72.0]	461	[14-72.0]

At five months of age, his calcium metabolism parameters and PTH levels were within normal ranges (Table 1). Over the first few years of life, the patient evolved with global developmental delay, behavioural abnormalities obesity and facial dysmorphisms. At examination, he presented trigonocephaly, coarse facial features, thick eyebrows, and frontal hemangioma and he didn’t show shortening of the fingers or toes. The patient exhibited behavioural alterations, initially marked by extreme inconsolability, emotional dysregulation, and self and hetero-aggressive behaviours. He also showed a delay in acquiring developmental milestones such as walking and language.

**Figure 1:**
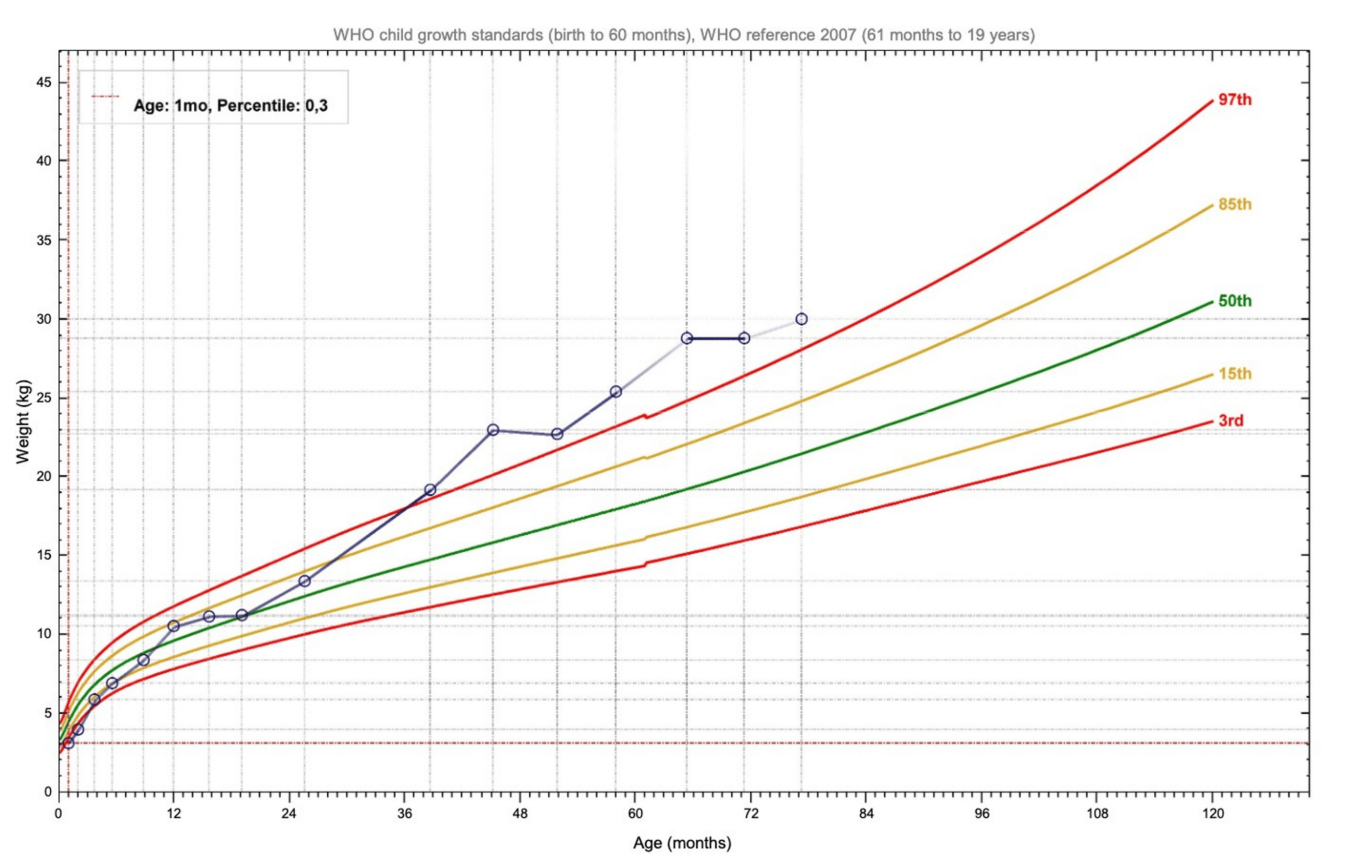
Patient's weight chart The curves were generated using WHO Anthroplus software (WHO, Geneva, Switzerland) [8].

**Figure 2:**
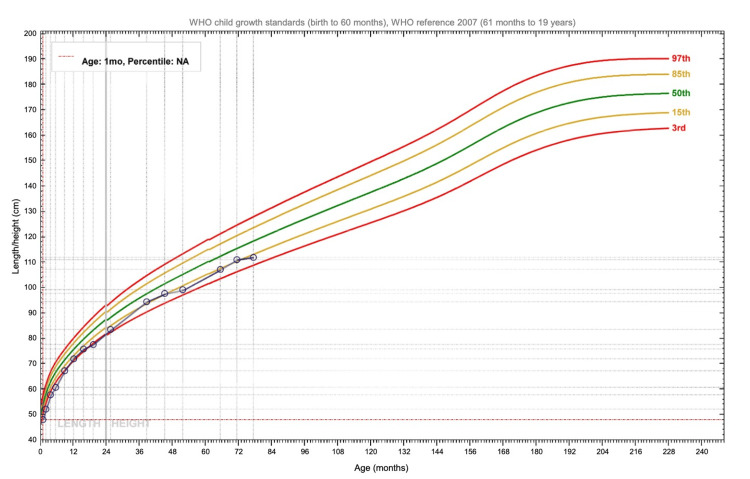
Patient's length/height chart The curves were generated using WHO Anthroplus software (WHO, Geneva, Switzerland) [8].

With regard to growth, his weight remained around the 85th percentile from age one until age two, after which it increased above the 97th percentile. His length stayed in the 3rd percentile until age two, then rose to the 15th percentile between ages three and four (Figures 1-2).

Given the importance of the combination of endocrinopathy, distinct facial dysmorphisms, and significant psychomotor developmental delay, the patient was referred for a comprehensive evaluation at the age of two by a multidisciplinary team of specialists in Development, Medical Genetics, Metabolic Diseases, and Child Psychiatry.

The neurodevelopmental evaluation confirmed global developmental delay, although vision, fine motor skills, and social adaptation were relatively preserved. The patient was enrolled in speech therapy, occupational therapy, and early intervention programs.

**Figure 3:**
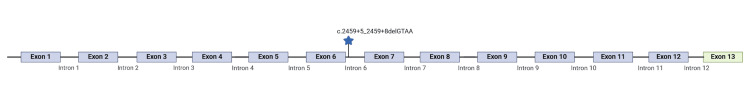
Graphic representation of the patient variant in the GNAS gene. The blue star indicates the variant identified in this study. Exons marked in blue correspond to those where maternally inherited pathogenic variants result in PHP1a phenotype. Exon marked in green correspond to the exon where maternally inherited pathogenic variants result in PHP subtype 1C phenotype.

Complementary investigations, including electroencephalogram, brain magnetic resonance imaging, echocardiogram, metabolic studies, array-CGH, and molecular testing for fragile X syndrome, all returned normal results. Whole-exome sequencing (WES) was performed to identify a heterozygous variant in the GNAS gene (c.2459+5_2459+8delGTAA), which was classified as a variant of uncertain significance (Figure 3). Segregation studies revealed the variant was maternally inherited.

This finding led to a suspicion of PHP, which was subsequently confirmed when repeated biochemical laboratory tests revealed hypocalcemia, hyperphosphatemia and elevated PTH levels of 461 pg/mL (Table 1). Calcitriol therapy was initiated.

**Figure 4:**
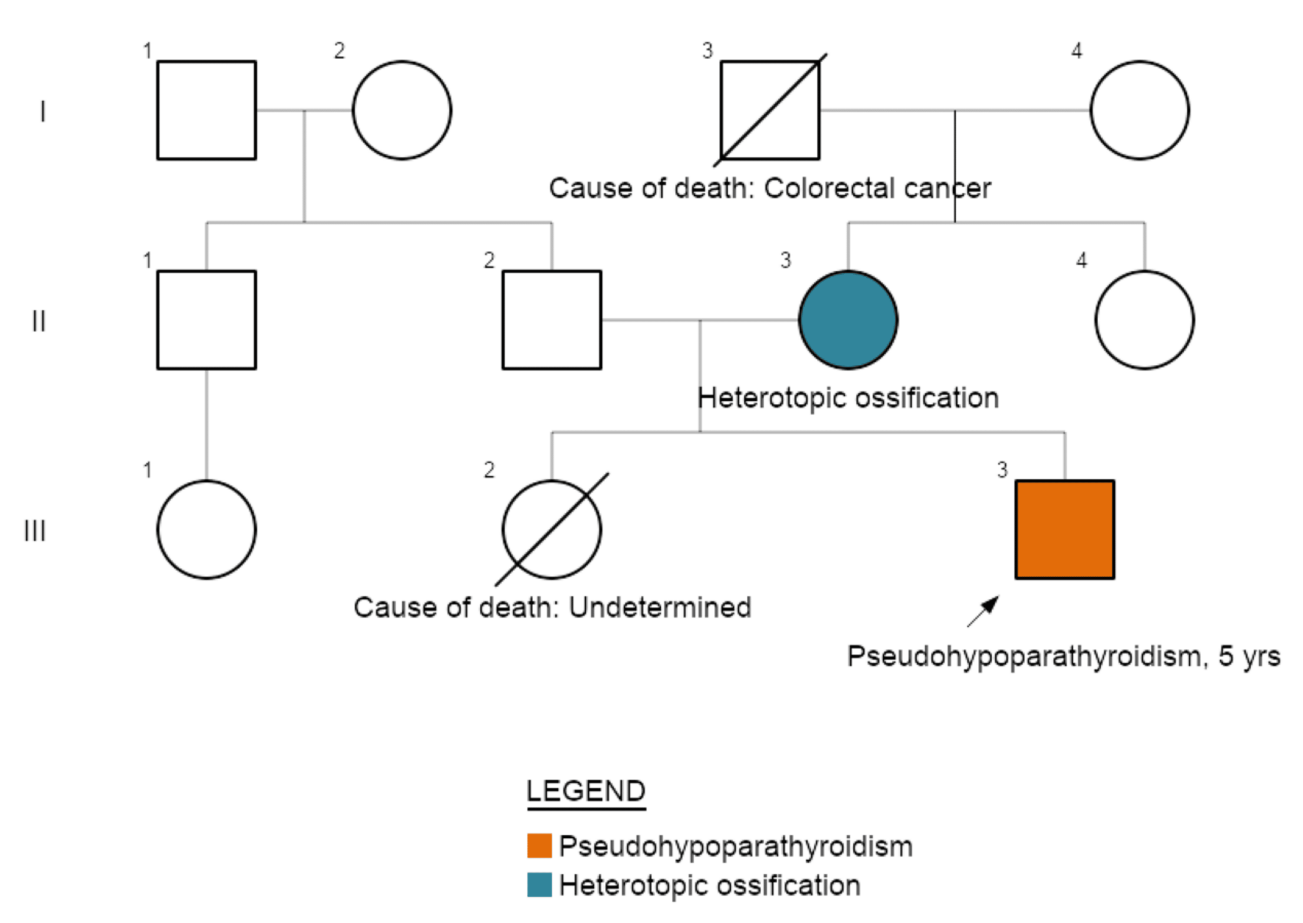
Familial Pedigree Chart

The patient’s mother examination revealed overweight and heterotopic ossification in deep connective tissues, confirmed by X-rays. Her biochemical tests showed absence of hormone resistance. These findings suggested progressive osseous heteroplasia, with no other findings. Additional segregation analysis showed that the healthy maternal grandmother did not carry this variant, and the maternal grandfather was already deceased, due to colorectal cancer (Figure 4). 

The identified variant is located in the intron 6 and affects the canonical acceptor splice-site nucleotide, leading to predicted exon skipping. This variant has previously been described as disease-causing for Albright hereditary osteodystrophy and co-segregates with the phenotype suggesting causality.

Currently, at six years of age, his height is at the 15th percentile and the patient remains under multidisciplinary care. From an endocrinologic perspective, he is stable on levothyroxine and calcitriol therapy. Neurodevelopmentally, the patient shows cognitive progress but continues to experience intense emotional episodes.

## Discussion

**Figure 5:**
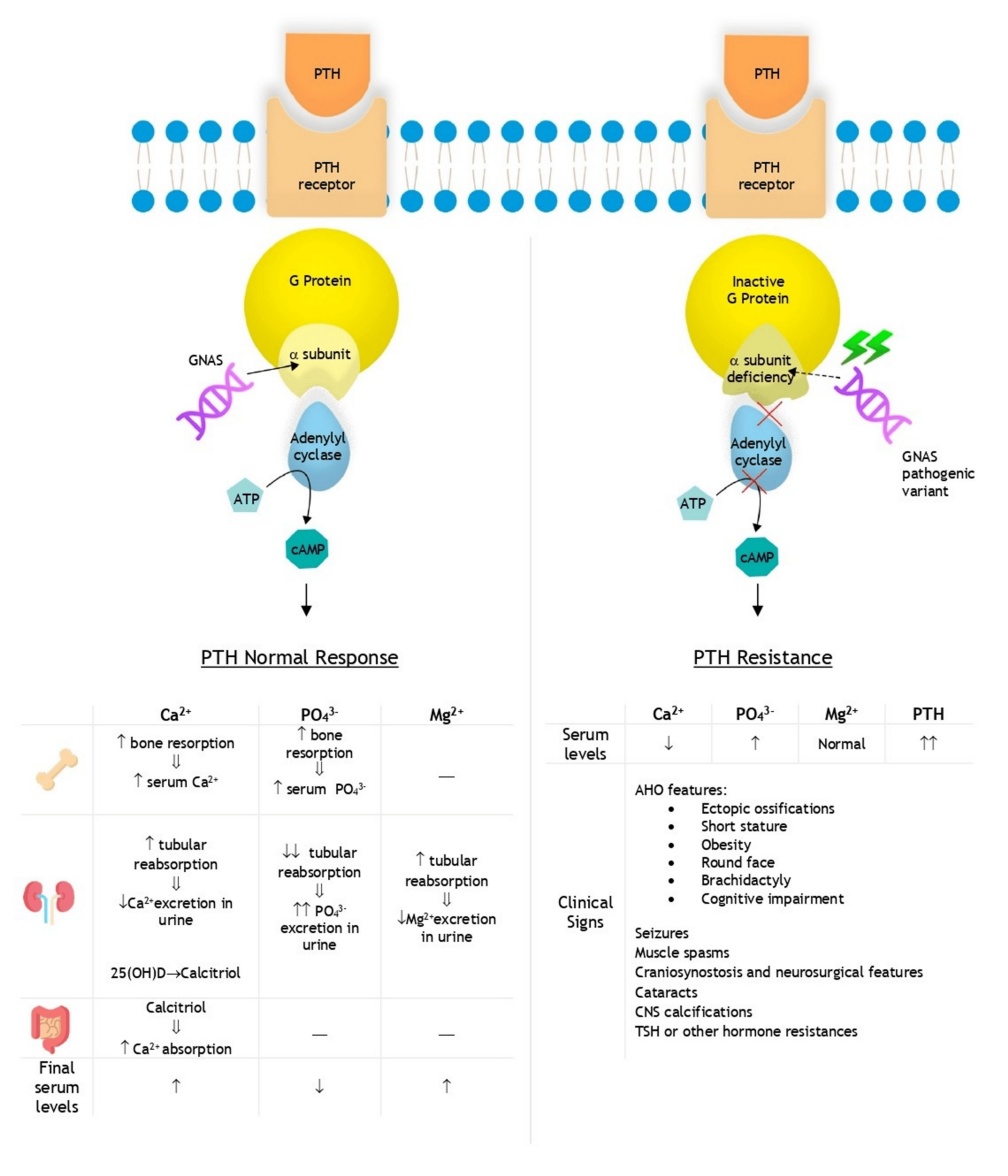
PTH signaling pathway and its effects: normal response vs. PTH resistance in pseudohypoparathyroidism The diagram illustrates the molecular mechanism of PTH action under normal conditions (left) and in the presence of PTH resistance (right), as seen in disorders like pseudohypoparathyroidism. On the left, PTH binds to its receptor, activating the G protein and stimulating adenylyl cyclase, which increases cAMP production and triggers physiological responses in target organs, leading to regulated serum levels of Ca²⁺, PO₄³⁻, and Mg²⁺. The table on the left outlines PTH’s effects on bone, kidneys, and the gastrointestinal tract, detailing the resulting changes in serum calcium, phosphate, and magnesium when there is a normal response to PTH. On the right, due to pathogenic variants in the GNAS, resulting in an α-subunit deficiency of the G protein, the stimulation of adenylyl cyclase is impaired, preventing the subsequent increase in cAMP production. The disruption of this signaling pathway leads to PTH resistance, resulting in altered serum electrolyte levels (↓ Ca²⁺, ↑ PO₄³⁻) and elevated PTH concentrations. This resistance manifests clinically with features of AHO—including ectopic ossifications, short stature, obesity, round face, brachydactyly, and cognitive impairment—as well as seizures, muscle spasms, CNS calcifications, and possible resistance to other hormones, such as TSH. 25(OH)D: 25-hydroxyvitamin D; AHO: Albright hereditary osteodystrophy; ATP: adenosine triphosphate; Ca²⁺: calcium; cAMP: cyclic adenosine monophosphate; CNS: central nervous system; Mg²⁺: magnesium; PO₄³⁻: phosphorus; PTH: parathyroid hormone; TSH: thyroid-stimulating hormone. Note: This image was created by the authors for this study.

PTH resistance is the hallmark of pseudohypoparathyroidism (PHP) [5], resulting in hypocalcemia and hyperphosphatemia [1] due to impaired cAMP-dependent signaling mediated by the Gsα protein (Figure 5) [9]. Unlike hypoparathyroidism, which is characterized by deficient PTH production, PHP is distinguished by elevated PTH levels despite normal vitamin D, magnesium, and renal function [5].

PHP is classified into five subtypes, PHP1A and PHP1B being the main two, with overlapping clinical features. PHP1A, the most common subtype, is associated with AHO, a condition characterized by short stature, brachydactyly, obesity, cognitive impairment, and heterotopic ossifications [1-3,5,9]. Newborns with PHP1A often present nonspecific features, but symptoms such as obesity, growth failure, brachydactyly, and hypocalcemia-related manifestations typically emerge as they age, leading to diagnosis. In contrast, PHP1B primarily involves PTH resistance without prominent AHO features. Patients with PHP1B may exhibit mild brachydactyly and, less frequently, TSH resistance, but they rarely present with ossifications or cognitive deficits [2]. Early-onset obesity is common in both subtypes, though more severe in PHP1A, and may serve as an early diagnostic clue [5,10].

The disease phenotype in PHP varies with the parental origin of the genetic alteration. PHP1A results from maternal heterozygous GNAS pathogenic variants in exons 1-12, whereas PHP1B is caused by loss of methylation in exon A/B, due to heterozygous deletion of STX16 or regulatory elements in the GNAS complex locus [3,5]. In contrast, pseudopseudohypoparathyroidism (PPH) results from paternal GNAS pathogenic variants. PPH patients may exhibit AHO features but lack hormone resistance, obesity, ionic imbalances, and neurodevelopmental issues seen in PHP1A. This genetic variability underscores the importance of molecular testing for accurate diagnosis, guiding management, preventing complications, and enabling genetic counseling [2,5].

In this case, the patient’s initial diagnosis of CH through neonatal screening is explained by thyroid hormone resistance, a feature often evident at birth in PHP [5]. Mildly elevated TSH levels with normal total T4 and fT4 in the lower normal range were consistent with mild TSH resistance, commonly seen in PHP [2]. At five months of age, calcium, phosphorus, magnesium and PTH levels were within normal ranges. In PHP, endocrine features gradually emerge over time, with mild hypothyroidism often being the earliest indication of hormone resistance [3]. PTH resistance usually develops later in childhood, [5,11] which accounts for the lack of immediate clinical signs pointing to an alternative diagnosis. There is no reliable biomarker for PHP at birth, as PTH, calcium, and phosphorus levels may be within the normal range, making it almost impossible to identify suggestive alterations at this stage.

Despite effective management of CH, the patient displayed syndromic features, developmental delay, behavioral changes, and obesity by age three. These findings were inconsistent with well-controlled CH, prompting further investigation and referral to the medical genetics department. This aligns with expert recommendations to consider PHP1A in children with treated CH who develop excessive weight gain and persistent neurodevelopmental delay, despite adequate thyroid hormone replacement [2,5].

Genetic testing identified a maternal heterozygous GNAS variant. Laboratory re-evaluation confirmed PHP1A with findings of hypocalcemia, hyperphosphatemia, and elevated PTH levels, alongside normal magnesium levels. While hallmark AHO features such as brachydactyly and heterotopic ossifications were absent, syndromic facial features, early-onset obesity, and cognitive impairments supported the PHP diagnosis. The absence of overt AHO features may delay clinical suspicion or might suggest PHP1B. However, the presence of TSH resistance and cognitive alterations aligned more closely with PHP1A [1,5]. Genetic analysis was essential in establishing an accurate diagnosis; without it, the clinical presentation would not have been immediately linked to this condition.

Management of PHP extends beyond calcium and active vitamin D supplementation to include regular monitoring of PTH, 25-OH vitamin D, and calcium levels, as well as assessing growth and endocrine deficits. In this case, the patient’s normal growth progression delayed the need for growth hormone (GH) resistance studies. However, regular evaluations of growth, pubertal stages and skeletal maturation remain critical, with recombinant human GH therapy considered if deficiency arises. The efficacy of pubertal blockers in increasing final height in PHP patients is uncertain [1,5]. Early dietary interventions for obesity and neuropsychological assessments at diagnosis are essential. Comprehensive care should also include clinical and radiological examinations for brachydactyly, and screening for sleep disorders, cataracts, and dental problems [1,5].

Previous cases of PHP1A have been documented, some sharing the initial diagnosis of CH [6,12-14]. Most of these cases present with classic AHO features, [6,9,12-18] facilitating clinical suspicion and diagnosis. However, our case highlights the challenges of recognizing PHP1A in early childhood, where the disease may present without the hallmark AHO features that can gradually emerge with growth. Most previously reported cases were diagnosed after the age of nine or in adult life [12,13,15,17], significantly later than our patient. This discrepancy in age at diagnosis may be explained by the absence of AHO features in our case, which could have led to a missed or even later diagnosis. The average age of diagnosis for PHP1A is around seven years when PTH resistance and hypocalcemia become apparent [3]. Individuals with milder manifestations may not receive a diagnosis until their third decade of life [3]. Therefore, the recognition of dysmorphic features, early-onset obesity, and developmental delay in this patient should serve as red flags for further investigation in those with well-managed CH.

Rare cases with earlier diagnosis have relied on specific findings such as osteoma cutis [6], hypocalcemia [14], or family history [16], which led to earlier identification of PHP. Studies have also highlighted the genetic complexity of the condition, including novel GNAS variants and variable intrafamilial expression, emphasizing the importance of molecular testing [9,13,16-18].

Recognizing PHP1A in early childhood is crucial for early intervention and preventing complications. However, the diagnosis can be challenging, particularly in the absence of classic AHO features and biochemical changes. Careful monitoring of growth, physical features, and developmental progress is key to maintaining clinical suspicion for this condition. This case underscores the importance of considering PHP1A, even in the absence of hypocalcemia and most typical AHO features, particularly in children with early-onset obesity, neurodevelopmental delays, dysmorphic features and thyroid hormone resistance. In such cases, more frequent evaluations throughout childhood could be considered, including monitoring calcium, phosphate, and PTH levels, which could theoretically aid in the early detection of biochemical changes associated with PTH resistance. Nevertheless, specialized and early genetic testing is crucial for accurate molecular diagnosis, enabling phenotype prediction, tailored management, and improved patient outcomes [5].

## Conclusions

PHP presents a diagnostic challenge due to its varied clinical manifestations and underlying genetic variations. Although rare, studying this condition offers valuable insights into hormone resistance mechanisms. Early identification and comprehensive clinical assessment, supported by genetic analysis, are essential for effective management. Identifying specific genetic mutations can help clinicians differentiate PHP from similar disorders, leading to earlier diagnosis and more personalized treatment strategies.

This case illustrates the diagnostic difficulties associated with PHP, especially when typical signs such as hypocalcemia or AHO features are absent. It also emphasizes the importance of a collaborative, multidisciplinary approach and the critical role of genetic evaluation in securing an accurate, early diagnosis and differentiating between the subtypes of PHP and similar conditions. These factors highlight the need for increased clinical awareness and tailored management plans to improve patient outcomes.
